# Combination of dasatinib and curcumin eliminates chemo-resistant colon cancer cells

**DOI:** 10.1186/1750-2187-6-7

**Published:** 2011-07-20

**Authors:** Jyoti Nautiyal, Shailender S Kanwar, Yingjie Yu, Adhip PN Majumdar

**Affiliations:** 1 Veterans Affairs Medical Center, Wayne State University, Detroit, MI 48201, USA; 2 Karmanos Cancer Institute, Wayne State University, Detroit, MI 48201, USA; 3 Department of Internal Medicine, Wayne State University, Detroit, MI 48201, USA

## Abstract

Metastatic colorectal cancer remains a serious health concern with poor patient survival. Although 5-Fluorouracil (5-FU) or 5-FU plus oxaliplatin (FOLFOX) is the standard therapy for colorectal cancer, it has met with limited success. Recurrence of the tumor after chemotherapy could partly be explained by the enrichment of the chemo-resistant sub-population of cancer stem cells (CSCs) that possess the ability for self-renewal and differentiation into different lineages in the tumor. Therefore development of therapeutic strategies that target CSCs for successful treatment of this malignancy is warranted. The current investigation was undertaken to examine the effectiveness of the combination therapy of dasatinib (a Src inhibitor) and curcumin (a dietary agent with pleiotropic effect) in inhibiting the growth and other properties of carcinogenesis of chemo-resistant colon cancer cells that are enriched in CSCs sub-population. Remnants of spontaneous adenomas from APC*^Min +/- ^*mice treated with dasatinib and/or curcumin were analyzed for several cancer stem cell markers (ALDH, CD44, CD133 and CD166). Human colon cancer cells HCT-116 (p53 wild type; *K-ras *mutant) and HT-29 (p53 mutant; *K-ras *wild type) were used to generate FOLFOX resistant (referred to as CR) cells. The effectiveness of the combination therapy in inhibiting growth, invasive potential and stemness was examined in colon cancer CR cells. The residual tumors from APC*^Min +/- ^*mice treated with dasatinib and/or curcumin showed 80-90% decrease in the expression of the CSC markers ALDH, CD44, CD133, CD166. The colon cancer CR cells showed a higher expression of CSCs markers, cell invasion potential and ability to form colonospheres, compared to the corresponding parental cells. The combination therapy of dasatinib and curcumin demonstrated synergistic interactions in CR HCT-116 and CR HT-29 cells, as determined by Calcusyn analysis. The combinatorial therapy inhibited cellular growth, invasion and colonosphere formation and also reduced CSC population as evidenced by the decreased expression of CSC specific markers: CD133, CD44, CD166 and ALDH. Our data suggest that the combination therapy of dasatinib and curcumin may be a therapeutic strategy for re-emergence of chemo-resistant colon cancer by targeting CSC sub-population.

## Background

Colorectal cancer, the third most common cancer affecting men and women equally [1], remains a huge health concern. It is the second most common cause of cancer-related deaths in the United States and other developed countries. Although with early detection and surgical resection, the 5-year survival rate can reach 90%, nearly 50% of patients with colorectal carcinoma develop recurrent disease [2,3]. Most of the colon cancer deaths results from the metastatic spread of chemotherapy-resistant cells to the liver and other organs [4] and thus, metastasis remains a poor prognostic indicator [5].

Over the last decade, there has been a growing body of evidence that support the concept of cancer stem cell (CSC) model as an explanation for the initiation, progression and recurrence of cancer. Epithelial cancers including colorectal cancer are now believed to be diseases driven by a minor subpopulation of self renewing cancer stem cells (CSCs). CSCs also have the potential to invade and form distant metastasis [6-10]. Biologically distinct and relatively rare populations of tumor-initiating cells or CSCs have been detected by several methods and markers established in a variety of cancers, including the colon [11-13]. Furthermore, CSCs are known to show resistance to a number of conventional chemotherapies and thus play a significant role in recurrence of primary cancers. Most of the conventional treatment regimen target the non-CSCs population of the tumor and fail to eliminate the CSCs [8,14]. The remaining chemotherapy-resistant CSCs lead to chemotherapy-refractory tumor, and may explain the difficulty in complete eradication of cancer and/or recurrence. Therefore, development of therapeutic strategies that specifically target CSCs is warranted in reducing the risk of relapse and metastasis.

5-Fluorouracil (5-FU) or 5-FU plus oxaliplatin (FOLFOX) remains the mainstay of colorectal cancer chemotherapeutics. Although these chemotherapeutic regimens produce a response in majority of the cases, virtually all the responses are incomplete and emergence of resistance with recurrence of the cancer is universal. There is also a cost of additional toxicities, some of which are even fatal. Therefore, validation of a non-toxic agent that could improve upon the current chemotherapeutic regimen(s) would be highly desirable. In an attempt to develop an effective treatment strategy, a combination therapeutic regimen comprising of dasatinib and curcumin (diferuloylmethane), was therefore tested for its efficacy in inhibiting growth and eliminating the CSCs in chemo-resistant colon cancer cells.

Dasatinib is a highly potent inhibitor of Src kinases and Abl kinases [15,16] and is currently employed for imatinib resistant CML and (Ph+) ALL treatment [15,16]. In addition to hematological malignancies, dasatinib has been shown to be effective in solid tumors and demonstrate multiple effects like inhibition of cellular growth, migration and invasion [15-17]. Our recent studies demonstrate that dasatinib regulates growth of breast cancer cells by modulating EGFR signaling [18]. Also, since multiple signaling pathways are deregulated in carcinogenesis, several therapeutic regimens that target multiple signaling are being investigated and developed. In this context, we have recently shown that dasatinib act synergistically with the pan-erbB inhibitor EBIP (ErbB inhibitory protein) as well as with curcumin in inhibiting several processes of carcinogenesis in breast and colon cancers [18,19]. The combination therapies were found to be highly effective in inhibiting cellular growth, colony formation, extracellular matrix invasion and attenuation of various signaling pathways [18,19].

Curcumin, the major active ingredient of turmeric, has been shown to inhibit chemically induced carcinogenesis in the skin, forestomach and colon when administered during initiation and/or post-initiation phases [20-23]. Development of azoxymethane-induced preneoplastic and neoplastic lesions of the colon is also inhibited in experimental animals fed a diet containing curcumin [24,25]. Curcumin has been shown to suppress various stages of colon carcinogenesis with no discernable toxicity [26,27] and prevent adenoma development in the intestinal tract of Min^+/- ^mice, a model of human familial adenomatous polyposis [28]. Furthermore, in a Phase I clinical trial, curcumin was shown to be effective in inhibiting tumor growth [29].

Herein, we demonstrate that curcumin synergizes with dasatinib to inhibit the growth of FOLFOX-resistant colon cancer cells that are highly enriched in CSCs. The combination therapy is also effective in inhibiting cell invasion and colonosphere formation, the latter of which is considered to be surrogate for tumor.

## Methods and materials

### Tissue procurement

Remnants of spontaneous adenomas from APC*^Min +/- ^*mice treated with dasatinib and/or curcumin were obtained from our previous study [30]. In this study we have demonstrated the superior efficacy of the combination therapy in inhibiting the formation as well as inducing the regression of spontaneous adenomas in APC*^Min +/- ^*mice than either agent alone. The tissues were processed for RNA isolation and analyzed for mRNA expression of various CSC markers.

### Cell lines and cell culture

Human colon cancer HCT-116 p53 wild type (*p53 *wild type; *K-ras *mutant), HT-29 (*K-ras *wild type; *p53 *mutant) and their FOLFOX resistant cell lines were used to investigate the efficacy of combined therapy of dasatinib and curcumin. HCT-116 and HT-29 cells were obtained from American Type Culture Collection (ATCC, Rockville, MD). The cells were maintained in tissue culture flasks in Dulbecco's modified Eagle medium (DMEM) in a humidified incubator at 37°C in an atmosphere of 95% air and 5% CO_2_. The cell culture medium was supplemented with 5% FBS and 1% antibiotic/antimycotic. FOLFOX resistant cell lines were generated in our laboratory (as reported previously) [31]. Briefly, HCT-116 or HT-29 cells were incubated with a clinically relevant dose of FOLFOX (25 μM 5-FU and 0.625 μM oxaliplatin) for one week. The adherent cells, which survived the FOLFOX insult, were subjected to trypsin/EDTA treatment and allowed to grow in normal DMEM for 2 weeks. The surviving cells were then split and gradually exposed to increasing doses of FOLFOX to a maximal concentration of 250 μM 5-FU and 6.25 μM oxaliplatin for 2-3 weeks for each treatment period. Finally, the chemo-resistant cells were maintained in normal culture medium containing FOLFOX (50 μM 5-FU + 1.25 μM oxaliplatin). The medium was changed three times a week and the cells were passaged using trypsin/EDTA.

### Growth inhibition assay and analysis of interaction between curcumin and dasatinib

Inhibition of cell growth in response to dasatinib and or curcumin was examined by 3-(4,5-dimethyl-thiazol-2yl)-2,5-diphenyl-tetrazolium bromide (MTT) assay as described previously [32]. Briefly, 5,000 cells/well were seeded into 96-well culture plates and treated with different doses of dasatinib and/or curcumin for 72 h. All CR cells were exposed to 50 μM 5-FU and 1.25 μM oxaliplatin with or without (control) dasatinib and/or curcumin. This treatment strategy was utilized in all subsequent experiments. The doses of dasatinib and curcumin for combination were chosen in fixed-ratio increments. The fraction of cells affected (Fa) by various treatments as determined by MTT assay, was utilized to generate dose response curves for dasatinib, curcumin and the combination therapy by employing Calcusyn software (Biosoft, Ferguson, MO). Further, Combination Indices (CI) were produced by Calcusyn software that utilizes the methodology applied by Chou and Talalay for formal synergy analyses [33]. This method utilizes a multiple drug-effect equation derived from enzyme kinetics model in which the output is represented as combination indices (CI) and/or isobologram analysis. Calcusyn software defines synergy when CI value is < 1. Synergy was defined based on terminology of Chou [34]. Based on CI values, the extent of synergism/antagonism may be determined. In brief, CI values between 0.9 and 0.85 would suggest a moderate synergy, whereas those in the range of 0.7 to 0.3 are indicative of clear synergistic interactions between the drugs. On the other hand, CI values in the range of 0.9 to 1.10 would suggest a near additive effect. All assays were performed in quadreplicates.

### Dose Reduction Index (DRI) determination

DRI is the measure of fold-decrease of individual agent when used in synergistic combination to achieve a given effect level compared with the doses of each drugs alone. The Calcusyn software was employed to calculate the DRI for dasatinib in CR cancer cell lines. The data generated from growth inhibition assays (Fa values for different combination doses) was utilized to determine DRIs. 

### Isolation of RNA and quantitative Polymerase Chain Reaction (PCR) Analysis

Total RNA was extracted from parental and chemo-resistant HCT-116 cells using Trizol reagent according to the manufacturer's instructions. RNA concentration was measured spectrophotometrically at an optical density of 260 nm. Quantitative reverse transcription-polymerase chain reaction (RT-PCR) was performed using the GeneAmp RNA PCR Kit (Applied Biosystems, Foster City, CA). Briefly, 1 μg of purified RNA was reverse-transcribed and the transcribed RNA was diluted five times for quantitative PCR amplification of various cancer stem cell markers. Five microliters of complementary DNA products was amplified with SYBR Green Quantitative PCR Master Mix (Applied Biosystems). Table 1 provides the list and sequences of the mouse and human specific primers used in the study. Reactions were carried out in Applied Biosystems 7500 Real-Time PCR System as described previously by Yu et al. [31]. The quantitation of the marker gene was normalized to amplification of β-actin and subsequently expressed as relative to untreated control.

**Table 1:** List of different PCR primers used in the study

Gene	Specificity	Direction	Primer Sequence
CD 44	Mouse	Forward	ctccagacaaccaccaggat

CD 44	Mouse	Reverse	tgtggggtctcctcttcatc

CD 133	Mouse	Forward	tcaaagggacccagaaactg

CD 133	Mouse	Reverse	gccttgttcttggtgttggt

CD 166	Mouse	Forward	ctcgttgctggtgtcgtcta

CD 166	Mouse	Reverse	tccaatccgctcctctctta

ALDH 1a	Mouse	Forward	gggctgacaagattcatggt

ALDH 1a	Mouse	Reverse	ggaaaattccaggggatgat

Actin	Mouse	Forward	agatctggcaccacaccttc

Actin	Mouse	Reverse	ggggtgttgaaggtctcaaa

CD 44	Human	Forward	aaggtggagcaaacacaacc

CD 44	Human	Reverse	actgcaatgcaaactgcaag

CD 133	Human	Forward	accgactgagacccaacatc

CD 133	Human	Reverse	ggtgctgttcatgttctcca

CD 166	Human	Forward	tagcaggaatgcaactgtgg

CD 166	Human	Reverse	cgcagacatagtttccagca

ALDH 1a	Human	Forward	gttgtcaaaccagcagagca

ALDH 1a	Human	Reverse	ctgtaggcccataaccagga

Actin	Human	Forward	cccagcacaatgaagatcaa

Actin	Human	Reverse	acatctgctggaaggtggac

ALDH1a represents ALDH1.

### Flow Cytometric Analyses for cancer stem cell markers

Parental (control) and chemo-resistant colon cancer cells were subjected to direct immunofluorescence staining followed by flow cytometric analyses. Briefly, the cells were harvested and washed with PBS. Two million cells were suspended in 90 μl of PBS containing 0.5% BSA for 10 minutes at room temperature followed by the addition of 10 μl of PerCP cy5.5 fluorescent dye conjugated to CD44 and/or phyco-erythrin conjugated CD-166 monoclonal antibody (Santa Cruz Biotechnology, Santa Cruz, CA) and incubated for 30 minutes in the dark, at room temperature. The samples were then washed and analyzed using a FACS DiVa (BD, San Jose, CA).

### Colonosphere Formation

To examine the effects of combination therapy on the formation of colonospheres by chemo-resistant cells, the ability of cell lines to form spheres in suspension was evaluated as described by Liu et al [24], with minor optimizations. Briefly, colonospheres were generated by incubating CR HCT-116 cells at a concentration of 250 cells per 100 μL in serum-free stem cell medium (SCM) containing DMEM/F12 (1:1) in 96-well ultra low-attachment plates (Corning Inc, Lowell, MA). The stem cell medium was supplemented with B27 (Life Technologies, Gaithersburg, MD), 20 ng/ml EGF (Sigma, St Louis, MO), 10 ng/ml fibroblast growth factor (Sigma), and antibiotic/antimycotic. After 24 h of cell seeding, dasatinib(1 μM) and curcumin(10 μM) were added to each well. The colonospheres formed in 10 days were photographed for five to six microscopic fields under a 10× objective. The colonospheres were further evaluated for their size by measuring the widest area of the sphere.

### Invasion assay

Invasion assay was performed as described by Nautiyal et al [32] using a colorimetric assay from the Chemicon International Inc. (Temecula, CA, USA). The kit utilizes ECMatrix™, a reconstituted basement membrane matrix of proteins. In brief, 5,000 CR HCT-116 cells were seeded in the insert with or without dasatinib (1 μM) and curcumin (10 μM), subsequently incubated at 37°C for 72 h. The inserts contained an 8 μm pore size polycarbonate membrane covered with a thin layer of EMatrix™. The ECM layer occluded the membrane pores, blocking the non-invasive cells from migrating through. However, invading cells migrated through the ECM layer and could be found attached to the bottom of polycarbonate layer. At the end of the incubation, non-invading cells were gently removed using a cotton-tipped swab from interior of the inserts. The invasive cells on the lower surface of inserts were stained with 500 μl of stain. For quantitation of invasive potential, the stained cells were solubilized with 200 μl of 10% acetic acid and a consistent volume of dye/solute mixture was read at 570 nm.

### Statistical Analysis

Unless otherwise stated, data were expressed as mean ± SD. Where applicable, the results were compared by using the unpaired, two-tailed Student t-test, as implemented by Excel 2007 (Microsoft Corp., Redmond, WA). p-value smaller than 0.05 was considered statistically significant.

## Results

### The combination of dasatinib and curcumin is effective in inhibiting cancer stem cell population in remnants of spontaneous adenomas

**Figure 1:**
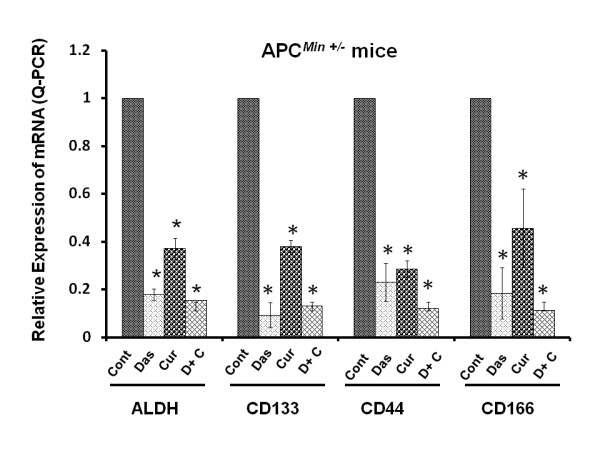
**Relative expression of various cancer stem cell markers in tumor remnants from APC*^Min +/- ^*mice treated with dasatinib and/or curcumin as determined by RT-PCR**. Tissue was procured from our previous study where Female *Min *mice (5 weeks; female C57BL/6J- APC*^Min +/- ^*) were treated with dasatinib (10 mg/kg body weight) and/or curcumin (250 mg/kg body weight). The treatment was given for five consecutive days per week for 4 weeks. At the end of respective treatments, the mice were euthanized and tumor remnants were obtained as described previously [30]. RNA isolated from the tissue was analyzed for expression of different CSCs specific markers.

Currently, CSCs are identified by specific surface epitopes. Colorectal CSCs cells were initially characterized by those expressing CD133 and subsequently by the expression of other surface markers such as CD44, CD166 and EpCAM/ESA (epithelial cell adhesion molecule/epithelial-specific antigen) [35]. More recently, aldehyde dehydrogenase 1 (ALDH1) which is a detoxifying enzyme [36] has been identified as a specific marker for normal and malignant human colonic stem cells [37]. ALDH1-positive cells, which are sparse and limited to the crypt bottom where stem cells reside, increase with progression of normal epithelium to adenoma to carcinoma [37]. We have recently reported that dasatinib in combination with curcumin is highly effective in inhibiting cellular growth and transformation properties *in vitro *and induces regression of over 90% of the spontaneous intestinal adenomas in APC^Min+/- ^mice [30]. This led to our interest in investigating the efficacy of the combined therapy in targeting CSCs that are implicated in the processes of chemo-resistance and recurrence of cancer. The initial experiment was carried out to evaluate the effectiveness of the combination therapy in inhibiting the growth of intestinal CSCs. For this, we utilized adenoma remnants derived from our previous study and examined the relative expression of various CSC specific markers. Figure 1 shows a marked 80-90% reduction in the expression of the CSC markers in response to the combination therapy, suggesting that the combination of dasatinib and curcumin is highly effective in reducing the cancer stem cell population in adenomas, the precursor for adenocarcinoma. Although dasatinib has met with limited success in solid tumors, our observation that the expression of CSC markers was greatly inhibited by dasatinib alone (Figure 1) provides a rationale for utilizing this drug in combination with more potent growth inhibitory agents like curcumin to achieve a superior anti-tumor response.

### Chemo-resistant colon cancer cells show increased expression of stem cell markers

**Figure 2:**
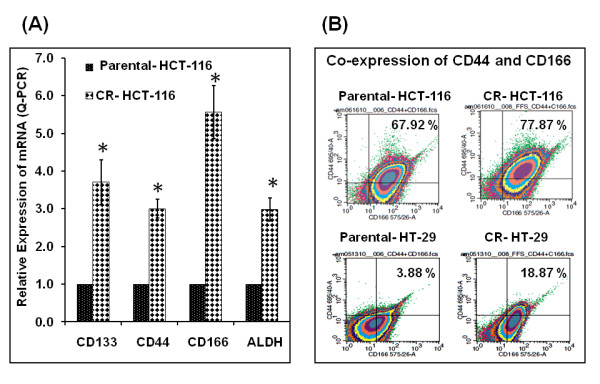
**(A) Relative expression of various cancer stem cell markers in chemo-resistant (CR) HCT-116 cells as determined by RT-PCR**; **(B) Sorting of anti-CD44 and anti-CD166 antibodies-tagged untreated (control) and CR HCT-116 and CR- HT-29 cells by flow cytometry**. Parental cells were maintained in DMEM, supplemented with 5% FBS and 1% antibiotic and antimycotic. The CR cells were continuously exposed to 50 μM 5-FU and 1.25 μM oxaliplatin.

In an attempt to characterize the chemo-resistant (CR) cells, the expression of CD133, CD44, CD166 and ALDH1 was quantitated relative to the parent cell line (Figure 2A). CR HCT-116 cells show higher expression of each of these cancer stem cell markers than the parental HCT-116 cells. CR colon cancer cells were also investigated for dual staining for surface markers that play significant role in adhesion, namely CD44 and CD166. Both CR HCT-116 and CR HT-29 cells show higher proportion of cells expressing the two CSC markers concurrently (Figure 2B). Although the population of dual staining cells was higher for HCT-116 cells, the relative increase was much higher in CR HT-29 cells (~6 time the parent cells) (Figure 2B). This suggests a higher population of CSCs in chemo-resistant cells. Therefore, we chose these cell lines as a model for testing our hypothesis.

### Combination therapy of dasatinib and curcumin is effective in inhibiting chemo-resistant colon cancer cells

**Figure 3:**
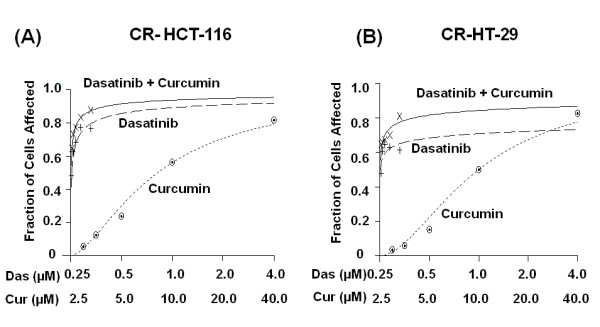
**Effects of dasatinib and/or curcumin on the growth of (A) CR HCT-116 and (B) CR HT-29 colon cancer cells: Growth as determined by MTT assay after 72-h incubation with incremental doses of dasatinib and/or curcumin**. All CR cells were exposed to 50 μM 5-FU and 1.25 μM oxaliplatin with or without (control) dasatinib and/or curcumin. This treatment strategy was utilized in all subsequent experiments. Dose response curves were generated for the drugs using Calcusyn 2.0 (Biosoft). All assays were performed in quadreplicates. A fractional effect (Fa) of 1 represents complete toxicity for the drug(s), whereas Fa value of "0" indicates no effect.

We have postulated that the combination therapy of curcumin and dasatinib would be a superior therapeutic strategy for chemo-resistant colorectal cancer. In order to test our hypothesis we first evaluated the interactions between dasatinib and curcumin in chemo-resistant (CR) colon cancer cells. We performed synergy analysis in CR HCT-116 and CR HT-29 cells, as described previously [30]. Dose response curves were generated for both agents in CR colon cancer cells using Calcusyn software (Biosoft, Ferguson, MO) (Figures 3A and 3B). In each CR cell line the combination therapy caused a greater growth inhibition than that achieved in response to a single agent (Figures 3A and 3B). As observed previously [30], the combination therapy was found to be more effective in HT-29 (p53 mutant) cells than HCT-116 cells.

The fraction of cells affected in response to each treatment was thus utilized to perform synergy analysis with Calcusyn software. The Combination Index (CI) as formulated by the software, revealed values of less than 1.0 indicating a synergistic interaction between the two agents at most of the dose combinations tested (Table 2). The results suggest that dasatinib and curcumin act synergistically to inhibit the growth of CR colon cancer cells. Since dasatinib and curcumin revealed synergistic interactions, the subsequent studies were performed with the combination of 1 μM dasatinib and 10 μM curcumin. Similar to our recent findings in parent cell lines [32], a higher synergy (lower CI value) was observed for lower dose combinations. Such synergistic interactions between various drugs provides an opportunity to reduce the concentrations of the individual drug(s) and thereby, reducing their associated toxicities.

**Table 2:** Synergy analysis for dasatinib and curcumin combination therapy in chemo-resistant colon cancer cells

COMBINATION THERAPY		COMBINATION INDEX (CI)	
**Dasatinib (μM)**	**Curcumin (μM)**	**CR HCT-116 (p53 wt)**	**CR HT-29 (p53 mutant)**

0.25	2.5	0.42	0.16

0.5	5.0	0.43	0.28

1.0	10.0	0.61	0.46

2.0	20.0	0.72	0.76

4.0	40.0	0.95	0.89

Combination indices < 1.0 are increasingly supra-additive, whereas values > 1.0 are increasingly less than additive.

### Combining curcumin with dasatinb is an effective way to reduce toxicity while retaining the therapeutic efficacy

Once the interaction between the two agents was found to be synergistic, we next sought to determine the DRI for dasatinib in CR colon cancer cells. Since combination index (CI) for both cell lines show strong synergy between dasatinib and curcumin, we performed DRI analysis for CR HCT-116 cells (Table 3). Our data show DRI values for dasatinib in the range from 13 to 25 for CR HCT-116 cells. The DRI data further demonstrated that when used in combination with dietary agent curcumin, dasatinib concentrations could be reduced significantly.

**Table 3:** Dose Reduction Index (DRI) analysis for dasatinb in chemo-resistant HCT-116 colon cancer cells

Drug Reduction Index for Dasatinib	
**Fa**	**DRI** **CR HCT-116 (p53 wt)**

0.25	13.37

0.50	18.19

0.75	24.76

DRI represents the order of magnitude (fold) of dose reduction obtained for specific Fa in combination settings as compared to each drug alone.

### The combination of dasatinib and curcumin inhibit colonosphere formation by chemo-resistant colon cancer cells

**Figure 4:**
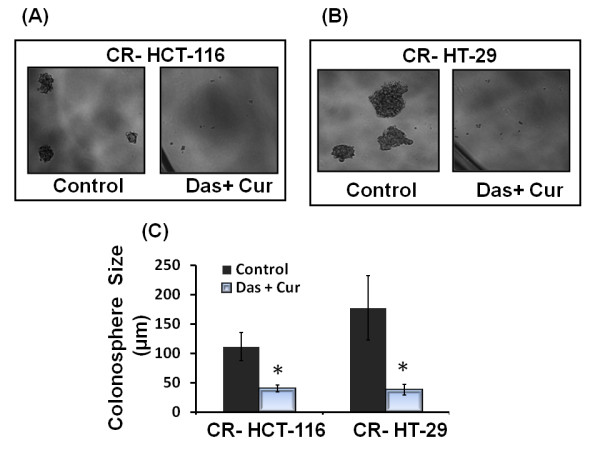
**Representative photograph showing formation of colonospheres by (A) CR HCT-116 and (B) CR HT-29 cells after incubating with dasatinib(1 μM), and curcumin (10 μM)**. Average size of colonospheres formed by CR HCT-116 and CR HT-29 in response to the combination therapy (C). The controls were incubated with FOLFOX (50 μM 5-FU + 1.25 μM oxaliplatin) containing medium only. *P < 0.01, compared with the corresponding control.

We next investigated whether the combination therapy would be effective in inhibiting the formation of colonosphere, a salient feature of cancer stem cells. The combination therapy was found to be highly effective in inhibiting the sphere forming potential of both CR HCT-116 and CR HT-29 cells (Figures 4A and 4B). At the end of 10-day experimental period we observed that while the control cells formed well-defined spheroids, the CR cells treated with the combination therapy showed significantly smaller spheres/spheroids (Figure 4C). The average sizes of colonospheres formed by the untreated CR HCT-116 and CR HT-29 cells were ~111 and 177 μm respectively (Figure 4C). In contrast, the average size of colonospheres formed in response to the combination therapy was 40 and 38 μm, respectively (Figure 4C). This suggests the current targeted therapy is highly effective in inhibiting the stemness properties of chemo-resistant colon cancer cells.

### The combination of dasatinib and curcumin effectively inhibit extracellular matrix invasion by chemo-resistant colon cancer cells

**Figure 5:**
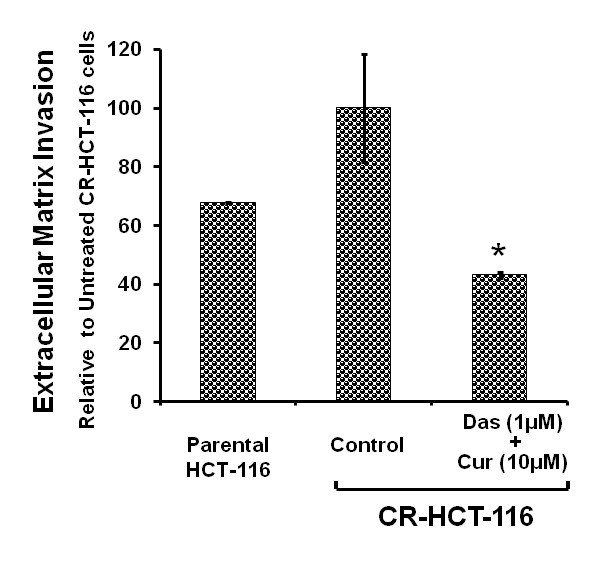
**Effects of dasatinib and curcumin on extra cellular invasion by CR HCT-116 colon cancer cells**. CR HCT-116 cells were allowed to invade in the presence or absence of dasatinib(1 μM), and curcumin (10 μM) for 3 days. The invading cells were stained with crystal violet and solubilized for quantitation. The controls represent the parental cells or CR HCT-116 cells that were incubated with FOLFOX (50 μM 5-FU + 1.25 μM oxaliplatin) containing medium only. *P < 0.01, compared with the corresponding control.

Chemo-resistant cells are thought to be more aggressive and have higher potential to invade through extracellular matrix leading to metastasis and spread of the primary malignancy than the parental cells. In view of this, we investigated the effectiveness of the current combination therapy in inhibiting the invasion potential of CR HCT-116 cells. Our results show that the chemo-resistant HCT-116 cells have higher potential to invade as compared to the corresponding parental cells and that they are highly susceptible to the combination therapy (Figure 5). This suggests that the combination therapy of the two targeted agents may be effective in targeting the chemo-resistant colon cancer.

### The combination of dasatinib and curcumin is effective in inhibiting cancer stem cell population in chemo-resistant colon cancer cells

**Figure 6:**
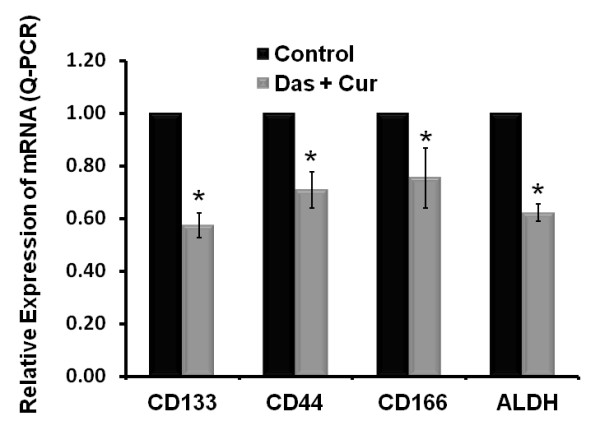
**Relative expression of various colon cancer stem cell markers in chemo-resistant HCT-116 cells in response to the exposure to dasatinib (1 μM), and curcumin (10 μM) for 3 days**. The controls represent the CR HCT-116 cells that were incubated with FOLFOX (50 μM 5-FU + 1.25 μM oxaliplatin) containing medium only. *P < 0.01, compared with the corresponding control.

Since the combination therapy is effective in inducing inhibition of growth, colonosphere formation and extracellular invasion, we next sought to test if this regimen would be effective in reducing the cancer stem cell population. The expression of CSC markers CD133, CD44, CD166 and ALDH1 was determined in the CR HCT-116 cells treated with the combination therapy. There was a 25-30% decrease in the expression of each of the CSC marker (Figures 2A and 2B). Although the CR cells displayed a higher expression of CSC markers and a higher proportion of cells showed co-expression of CD44 and CD166 (Figures 2A &2B), the combination therapy greatly decreased the expression of these proteins (Figure 6). These findings suggest that the combination therapy is highly effective in reducing the CSC sub-population in the chemo-resistant colon cancer and may be utilized as a CSC targeted therapy for elimination of recurrence of colon cancer.

## Discussion and Conclusion

Metastatic colorectal cancer remains incurable, indicating a poor prognosis and overall survival of about 2 years in surgically unresectable disease. FOLFOX is the standard therapy for colorectal cancer with limited success. Oxaliplatin, a platinum based chemotherapeutic agent form platinum-DNA adducts and interferes with DNA replication leading to cell death [38]. Development of resistance to FOLFOX is a common phenomenon leading to recurrence of the tumor. Src, a tyrosine kinase, that regulates diverse cellular processes, has been associated with various stages of cancer progression with an inverse co-relation with patient survival [39]. Enhanced Src activity has been reported in > 70% colon cancer, though the highest activity is demonstrated in metastasis [40,41]. More recently, Src kinase has been implicated in drug resistance [42] and is shown to be a modulator of sensitivity to oxaliplatin [43]. Pre-clinical evidence indicates that inhibition of Src renders the cancer cells susceptible to chemotherapies [44-46]. In light of these findings, investigations are being carried out to develop dasatinib, a highly potent Src inhibitor, as an adjuvant therapy for treatment of cancer along with inhibition of recurrence. However, administration of multiple therapeutic agents is often associated with additional toxicities which at times may be life threatening. Therefore, a combination with non-toxic dietary agent like curcumin is expected to provide a superior benefit. Curcumin, a phytochemical has been reported to have anti-tumor effects in various solid tumors [27]. Curcumin has been shown to augment the effect of a number of chemotherapeutic agents, including doxorubicin and vincristine and has been shown to enhance the cellular accumulation of these drugs leading to increased sensitivity of the drug-resistant cancer cells [47,48]. We have demonstrated that the combination of curcumin and FOLFOX causes a marked inhibition of growth of colon cancer cells [49]. More recently, we reported that curcumin is highly effective in sensitizing the FOLFOX surviving colon cancer cells [31]. The chemo-resistant HCT-116 cells show increased expression of several biomarkers of CSCs, a greater ability to form colonies and colonospheres, indicating an increase in the CSCs population [31]. We demonstrated that curcumin alone or in combination with FOLFOX markedly decreases CSCs, as determined by decreased expression of CSCs markers and colony as well as colonosphere formation [31].

In the current investigation, we utilized FOLFOX resistant cells derived from two colon cancer cell lines with different mutational status; HCT-116 p53 wild type (*p53 *wild type; *K-ras *mutant), HT-29 (*K-ras *wild type; *p53 *mutant). We have recently reported synergistic interaction between dasatinib and curcumin in parental HCT-116 and HT-29 cells [32]. Herein, we report similar synergistic effects on the chemo-resistant derivatives of these cells. Interestingly, as revealed by the combination indices, like the parent cells, CR HT-29 cells also show a higher susceptibility to the combination therapy than CR HCT-116 cells. This can be explained on the basis of recent findings of Kopetz et al. [43]. They demonstrated that in HT-29 cells, the expression of Src is greatly augmented in response to oxaliplatin, and thus the inhibition of Src by dasatinib imparts greater sensitivity to oxaliplatin. On the other hand, HCT-116 cells that did not demonstrate an increase in Src expression failed to show such synergy between the two agents. Our current finding supports the contention that cancer cells that are highly dependent on Src signaling will be more susceptible to Src-targeted combination therapy.

Development of dasatinib as a therapeutic agent has been hindered by the toxicities and associated adverse effects. Our data, for the first time, demonstrate that by using curcumin, dasatinib dose could be markedly reduced as evidenced by the DRI calculations. This is particularly significant in clinical situations where reduced toxicity towards the host is much sought after. These observations suggest that combining dietary agents to conventional chemotherapeutics is greatly beneficial in achieving better therapeutic effects.

Failure of current chemotherapies to eliminate CSCs is thought to be a major hurdle in treating colon cancer. CSCs possess the potential to self-renew as well as to differentiate into heterogenous population of cells within the tumor. They are currently identified by the expression of specific surface and cytoplasmic markers. Potential markers for colon CSCs include CD133, CD44, CD166 and ALDH1 [35]. Although the functional importance of CD133 remains to be resolved [50], CD44 is a transmembrane glycoprotein that has a unique cell adhesion function. CD44 plays a role in cancer cell migration as well as matrix adhesion and is involved in increased tumor growth [50,51]. CD166 is another adhesion molecule whose functional role is unclear. However, the expression of CD166 is found to be increased in primary adenocarcinomas and is associated with shorter patient survival [52]. Another marker with functional significance is aldehyde dehydrogenase 1 (ALDH1), which is a detoxifying metabolic enzyme, found to be associated with stem cell population [53]. In addition to being a potential cancer stem cell marker [37], ALDH1 is proposed to be associated with chemo-resistance of the subset of cells that show CD44 and CD166 dual positivity [54]. We have observed that the colon cancer cells that are continually exposed to FOLFOX lead to increased expression of CSC markers. Our current data demonstrate increased expression of CD133, CD44, CD166 and ALDH1 in chemo-resistant colon cancer cells. Dalerba et al. have reported that tumorigenicity is more specific and restricted to CSCs that express both CD44 and CD166 [35]. Interestingly, we have observed that CR HCT-116 as well as CR HT-29 cells contain a higher proportion of cells that express both CD44 and CD166 than the corresponding parental cell lines. Taken together the results show that, the chemo-resistant colon cancer cells are enriched in CSCs. We also report that the remnants of spontaneous adenomas from mice treated with dasatinib and/or curcumin showed 80-90% decrease in the expression of the CSC markers ALDH1, CD44, CD133, CD166.

Our current data demonstrate that the combination therapy is highly effective in inhibiting the processes of carcinogenesis as evidenced by decreased colonosphere formation by the chemo-resistant cells that are highly enriched in CSCs. CR HCT-116 cells also show a higher invasive potential and greater susceptibility to the combination therapy, suggesting that this treatment could be an effective therapeutic strategy for targeting chemo-resistant cancer cells. Furthermore, the combination treatment is highly effective in eliminating the CSCs population in the CR HCT-116 colon cancer cells. The expression of CSC markers is significantly reduced in response to the combination therapy. Considering the fact that the combination therapy is highly effective in inhibiting stem cell population in primary tumors as well as chemo-resistant colon cancer cells, our current observation suggests that the targeted combination regimen could be a superior strategy in eliminating CSCs and may inhibit the re-emergence of colon cancer. Additionally, incorporation of curcumin, a phytochemical will enable us to achieve greater benefit with reduced drug toxicities. This dietary agent which is well tolerated, could be added to food and taken on a long-term basis to either prevent primary tumor formation or tumor recurrence [55].

## List of abbreviations

5-FU: 5-Fluorouracil; ALDH1: aldehyde dehydrogenase 1; ATCC: American type culture collection; CI: combination indices; CML: chronic myelogenous leukemia; CSCs: cancer stem cells; CR: chemo-resistant; DMEM: Dulbecco's modified Eagle medium; EpCAM/ESA: epithelial cell adhesion/epithelial specific antigen; Fa: fraction of cells affected; FOLFOX: 5-FU plus oxaliplatin; MTT: 3-(4,5-dimethyl-thiazol-2yl)-2,5-diphenyl-tetrazolium bromide; PCR: Polymerase Chain Reaction; (Ph+) ALL: Philadelphia chromosome-positive acute lymphoblastic leukemia; RNA: ribo-nucleic acid; RT-PCR: Reverse transcription- polymerase chain reaction; SCM: stem cell media; SD: standard deviation.

## Competing interests

None of the following authors have any conflict of interest: JN, SSK, YY, APNM.

## Authors' contributions

JN carried out the experiments and wrote the manuscript. SSK helped in discussion and interpretation of the data. YY designed all the primers utilized in this manuscript. APNM, the principal investigator, was responsible for planning, designing, analysis of the data and overall supervision of the work and final preparation of the manuscript. All authors read and approved the final manuscript.

## Authors' information

Jyoti Nautiyal, PhD; Postdoctoral Research Fellow, Department of Internal Medicine and Veterans Affairs Medical Center, Wayne State University, Detroit, MI 48201, USA. E-mail: jyotinautiyal@gmail.com.

Shailender S. Kanwar, Ph.D.: Postdoctoral Research Fellow, Department of Internal Medicine and Veterans Affairs Medical Center, Wayne State University, Detroit, MI 48201, USA. E-mail: sskanwar@gmail.com

Yingjie Yu, M.D., Research Assistant Professor, Department of Internal Medicine and Veterans Affairs Medical Center, Wayne State University, Detroit, MI 48201, USA. E-mail: aa5142@wayne.edu

Adhip P.N. Majumdar, Ph.D., D.Sc.: Professor and Senior Research Career Scientist, Department of Internal Medicine, Veterans Affairs Medical Center. E-mail: a.majumdar@wayne.edu

## References

[B1] Jemal A Center MM Ward E Thun MJ Cancer occurrence Methods Mol Biol 2009 471 3 29 10.1007/978-1-59745-416-2_1 19109772

[B2] Andre T Boni C Mounedji-Boudiaf L Navarro M Tabernero J Hickish T Topham C Zaninelli M Clingan P Bridgewater J Tabah-Fisch I de Gramont A Oxaliplatin, fluorouracil, and leucovorin as adjuvant treatment for colon cancer N Engl J Med 2004 350 2343 2351 10.1056/NEJMoa032709 15175436

[B3] Neugut AI Lautenbach E Abi-Rached B Forde KA Incidence of adenomas after curative resection for colorectal cancer Am J Gastroenterol 1996 91 2096 2098 8855728

[B4] Welch JP Donaldson GA The clinical correlation of an autopsy study of recurrent colorectal cancer Ann Surg 1979 189 496 502 443905 10.1097/00000658-197904000-00027 PMC1397255

[B5] O'Connell JB Maggard MA Ko CY Colon cancer survival rates with the new American Joint Committee on Cancer sixth edition staging J Natl Cancer Inst 2004 96 1420 1425 10.1093/jnci/djh275 15467030

[B6] Dalerba P Clarke MF Cancer stem cells and tumor metastasis: first steps into uncharted territory Cell Stem Cell 2007 1 241 242 10.1016/j.stem.2007.08.012 18371356

[B7] Boman BM Huang E Human colon cancer stem cells: a new paradigm in gastrointestinal oncology J Clin Oncol 2008 26 2828 2838 10.1200/JCO.2008.17.6941 18539961

[B8] Jordan CT Guzman ML Noble M Cancer stem cells N Engl J Med 2006 355 1253 1261 10.1056/NEJMra061808 16990388

[B9] Dick JE Stem cell concepts renew cancer research Blood 2008 112 4793 4807 10.1182/blood-2008-08-077941 19064739

[B10] Wang JC Dick JE Cancer stem cells: lessons from leukemia Trends Cell Biol 2005 15 494 501 10.1016/j.tcb.2005.07.004 16084092

[B11] Al-Hajj M Wicha MS Benito-Hernandez A Morrison SJ Clarke MF Prospective identification of tumorigenic breast cancer cells Proc Natl Acad Sci USA 2003 100 3983 3988 10.1073/pnas.0530291100 12629218 PMC153034

[B12] Mizrak D Brittan M Alison MR CD133: molecule of the moment J Pathol 2008 214 3 9 10.1002/path.2283 18067118

[B13] O'Brien CA Pollett A Gallinger S Dick JE A human colon cancer cell capable of initiating tumour growth in immunodeficient mice Nature 2007 445 106 110 10.1038/nature05372 17122772

[B14] Dean M Fojo T Bates S Tumour stem cells and drug resistance Nat Rev Cancer 2005 5 275 284 10.1038/nrc1590 15803154

[B15] Araujo J Logothetis C Dasatinib: A potent SRC inhibitor in clinical development for the treatment of solid tumors Cancer Treatment Reviews 2010 36 492 500 10.1016/j.ctrv.2010.02.015 20226597 PMC3940067

[B16] Kim LC Rix U Haura EB Dasatinib in solid tumors Expert Opin Investig Drugs 2010 19 415 425 10.1517/13543781003592097 20113198

[B17] Serrels A Macpherson IRJ Evans TRJ Lee FY Clark EA Sansom OJ Ashton GH Frame MC Brunton VG Identification of potential biomarkers for measuring inhibition of Src kinase activity in colon cancer cells following treatment with dasatinib Mol Cancer Ther 2006 5 3014 3022 10.1158/1535-7163.MCT-06-0382 17148760

[B18] Nautiyal J Majumder P Patel BB Lee FY Majumdar AP Src inhibitor dasatinib inhibits growth of breast cancer cells by modulating EGFR signaling Cancer Lett 2009 8;283 2 143 51 19398150 10.1016/j.canlet.2009.03.035

[B19] Nautiyal J Yu Y Aboukameel A Kanwar SS Das JK Du J Patel BB Sarkar FH Rishi AK Mohammad RM Majumdar APN ErbB-Inhibitory Protein: A Modified Ectodomain of Epidermal Growth Factor Receptor Synergizes with Dasatinib to Inhibit Growth of Breast Cancer Cells Molecular Cancer Therapeutics 2010 9 6 1503 14 10.1158/1535-7163.MCT-10-0019 20515951 PMC2884079

[B20] Huang MT Lou YR Ma W Newmark HL Reuhl KR Conney AH Inhibitory effects of dietary curcumin on forestomach, duodenal, and colon carcinogenesis in mice Cancer Res 1994 54 5841 5847 7954412

[B21] Huang MT Ma W Yen P Xie JG Han J Frenkel K Grunberger D Conney AH Inhibitory effects of topical application of low doses of curcumin on 12-O-tetradecanoylphorbol-13-acetate-induced tumor promotion and oxidized DNA bases in mouse epidermis Carcinogenesis 1997 18 83 88 10.1093/carcin/18.1.83 9054592

[B22] Huang MT Smart RC Wong CQ Conney AH Inhibitory effect of curcumin, chlorogenic acid, caffeic acid, and ferulic acid on tumor promotion in mouse skin by 12-O-tetradecanoylphorbol-13-acetate Cancer Res 1988 48 5941 5946 3139287

[B23] Huang MT Wang ZY Georgiadis CA Laskin JD Conney AH Inhibitory effects of curcumin on tumor initiation by benzo[a]pyrene and 7,12-dimethylbenz[a]anthracene Carcinogenesis 1992 13 2183 2186 10.1093/carcin/13.11.2183 1423891

[B24] Rao CV Rivenson A Simi B Zang E Kelloff G Steele V Reddy BS Chemoprevention of colon carcinogenesis by sulindac, a nonsteroidal anti-inflammatory agent Cancer Res 1995 55 1464 1472 7882354

[B25] Rao CV Simi B Reddy BS Inhibition by dietary curcumin of azoxymethane-induced ornithine decarboxylase, tyrosine protein kinase, arachidonic acid metabolism and aberrant crypt foci formation in the rat colon Carcinogenesis 1993 14 2219 2225 10.1093/carcin/14.11.2219 8242846

[B26] Hanif R Qiao L Shiff SJ Rigas B Curcumin, a natural plant phenolic food additive, inhibits cell proliferation and induces cell cycle changes in colon adenocarcinoma cell lines by a prostaglandin-independent pathway J Lab Clin Med 1997 130 576 584 10.1016/S0022-2143(97)90107-4 9422331

[B27] Chauhan DP Chemotherapeutic potential of curcumin for colorectal cancer Curr Pharm Des 2002 8 1695 1706 10.2174/1381612023394016 12171541

[B28] Perkins S Verschoyle RD Hill K Parveen I Threadgill MD Sharma RA Williams ML Steward WP Gescher AJ Chemopreventive efficacy and pharmacokinetics of curcumin in the min/+ mouse, a model of familial adenomatous polyposis Cancer Epidemiol Biomarkers Prev 2002 11 535 540 12050094

[B29] Sharma RA Euden SA Platton SL Cooke DN Shafayat A Hewitt HR Marczylo TH Morgan B Hemingway D Plummer SM Pirmohamed M Gescher AJ Steward WP Phase I clinical trial of oral curcumin: biomarkers of systemic activity and compliance Clin Cancer Res 2004 10 6847 6854 10.1158/1078-0432.CCR-04-0744 15501961

[B30] Nautiyal J Banerjee S Kanwar SS Yu Y Patel BB Sarkar FH Majumdar AP Curcumin enhances dasatinib-induced inhibition of growth and transformation of colon cancer cells Int J Cancer 2011 128 951 961 10.1002/ijc.25410 20473900 PMC2939251

[B31] Yu Y Kanwar SS Patel BB Nautiyal J Sarkar FH Majumdar AP Elimination of Colon Cancer Stem-Like Cells by the Combination of Curcumin and FOLFOX Transl Oncol 2009 2 321 328 19956394 10.1593/tlo.09193 PMC2781082

[B32] Kanwar SS Nautiyal J Majumdar AP EGFR(S) inhibitors in the treatment of gastro-intestinal cancers: what's new? Curr Drug Targets 2010 11 682 698 10.2174/138945010791170851 20298154 PMC3915939

[B33] Chou TC Talalay P Quantitative analysis of dose-effect relationships: the combined effects of multiple drugs or enzyme inhibitors Adv Enzyme Regul 1984 22 27 55 6382953 10.1016/0065-2571(84)90007-4

[B34] Chou TC Theoretical basis, experimental design, and computerized simulation of synergism and antagonism in drug combination studies Pharmacol Rev 2006 58 621 681 10.1124/pr.58.3.10 16968952

[B35] Dalerba P Dylla SJ Park IK Liu R Wang X Cho RW Hoey T Gurney A Huang EH Simeone DM Shelton AA Parmiani G Castelli C Clarke MF Phenotypic characterization of human colorectal cancer stem cells Proc Natl Acad Sci USA 2007 104 10158 10163 10.1073/pnas.0703478104 17548814 PMC1891215

[B36] Magni M Shammah S Schiro R Mellado W Dalla-Favera R Gianni AM Induction of cyclophosphamide-resistance by aldehyde-dehydrogenase gene transfer Blood 1996 87 1097 1103 8562935

[B37] Huang EH Hynes MJ Zhang T Ginestier C Dontu G Appelman H Fields JZ Wicha MS Boman BM Aldehyde dehydrogenase 1 is a marker for normal and malignant human colonic stem cells (SC) and tracks SC overpopulation during colon tumorigenesis Cancer Res 2009 69 3382 3389 10.1158/0008-5472.CAN-08-4418 19336570 PMC2789401

[B38] Kelland L The resurgence of platinum-based cancer chemotherapy Nat Rev Cancer 2007 7 573 584 10.1038/nrc2167 17625587

[B39] Aligayer H Boyd DD Heiss MM Abdalla EK Curley SA Gallick GE Activation of Src kinase in primary colorectal carcinoma: an indicator of poor clinical prognosis Cancer 2002 94 344 351 10.1002/cncr.10221 11900220

[B40] Talamonti MS Roh MS Curley SA Gallick GE Increase in activity and level of pp60c-src in progressive stages of human colorectal cancer J Clin Invest 1993 91 53 60 10.1172/JCI116200 7678609 PMC329994

[B41] Termuhlen PM Curley SA Talamonti MS Saboorian MH Gallick GE Site-specific differences in pp60c-src activity in human colorectal metastases J Surg Res 1993 54 293 298 10.1006/jsre.1993.1046 7687314

[B42] Kopetz S Shah AN Gallick GE Src continues aging: current and future clinical directions Clin Cancer Res 2007 13 7232 7236 10.1158/1078-0432.CCR-07-1902 18094400

[B43] Kopetz S Lesslie DP Dallas NA Park SI Johnson M Parikh NU Kim MP Abbruzzese JL Ellis LM Chandra J Gallick GE Synergistic Activity of the Src Family Kinase Inhibitor Dasatinib and Oxaliplatin in Colon Carcinoma Cells Is Mediated by Oxidative Stress Cancer Research 2009 69 3842 3849 10.1158/0008-5472.CAN-08-2246 19383922 PMC2709758

[B44] Duxbury MS Ito H Zinner MJ Ashley SW Whang EE Inhibition of Src Tyrosine Kinase Impairs Inherent and Acquired Gemcitabine Resistance in Human Pancreatic Adenocarcinoma Cells Clinical Cancer Research 2004 10 2307 2318 10.1158/1078-0432.CCR-1183-3 15073106

[B45] George JA Chen T Taylor CC Src Tyrosine Kinase and Multidrug Resistance Protein-1 Inhibitions Act Independently but Cooperatively to Restore Paclitaxel Sensitivity to Paclitaxel-Resistant Ovarian Cancer Cells Cancer Research 2005 65 10381 10388 10.1158/0008-5472.CAN-05-1822 16288028

[B46] Griffiths GJ Koh MY Brunton VG Cawthorne C Reeves NA Greaves M Tilby MJ Pearson DG Ottley CJ Workman P Frame MC Dive C Expression of Kinase-defective Mutants of c-Src in Human Metastatic Colon Cancer Cells Decreases Bcl-xL and Increases Oxaliplatin- and Fas-induced Apoptosis Journal of Biological Chemistry 2004 279 46113 46121 10.1074/jbc.M408550200 15326164

[B47] Harbottle A Daly AK Atherton K Campbell FC Role of glutathione S-transferase P1, P-glycoprotein and multidrug resistance-associated protein 1 in acquired doxorubicin resistance Int J Cancer 2001 92 777 783 10.1002/ijc.1283 11351295

[B48] Verma SP Goldin BR Lin PS The inhibition of the estrogenic effects of pesticides and environmental chemicals by curcumin and isoflavonoids Environ Health Perspect 1998 106 807 812 9831541 10.1289/ehp.106-1533252 PMC1533252

[B49] Patel BB Sengupta R Qazi S Vachhani H Yu Y Rishi AK Majumdar AP Curcumin enhances the effects of 5-fluorouracil and oxaliplatin in mediating growth inhibition of colon cancer cells by modulating EGFR and IGF-1R Int J Cancer 2008 122 267 273 10.1002/ijc.23097 17918158

[B50] Du L Wang H He L Zhang J Ni B Wang X Jin H Cahuzac N Mehrpour M Lu Y Chen Q CD44 is of Functional Importance for Colorectal Cancer Stem Cells Clinical Cancer Research 2008 14 6751 6760 10.1158/1078-0432.CCR-08-1034 18980968

[B51] Aruffo A Stamenkovic I Melnick M Underhill CB Seed B CD44 is the principal cell surface receptor for hyaluronate Cell 1990 61 1303 1313 10.1016/0092-8674(90)90694-A 1694723

[B52] Weichert W Knosel T Bellach J Dietel M Kristiansen G ALCAM/CD166 is overexpressed in colorectal carcinoma and correlates with shortened patient survival J Clin Pathol 2004 57 1160 1164 10.1136/jcp.2004.016238 15509676 PMC1770486

[B53] Armstrong L Stojkovic M Dimmick I Ahmad S Stojkovic P Hole N Lako M Phenotypic characterization of murine primitive hematopoietic progenitor cells isolated on basis of aldehyde dehydrogenase activity Stem Cells 2004 22 1142 1151 10.1634/stemcells.2004-0170 15579635

[B54] Dylla SJ Beviglia L Park IK Chartier C Raval J Ngan L Pickell K Aguilar J Lazetic S Smith-Berdan S Clarke MF Hoey T Lewicki J Gurney AL Colorectal cancer stem cells are enriched in xenogeneic tumors following chemotherapy PLoS One 2008 3 e2428 10.1371/journal.pone.0002428 18560594 PMC2413402

[B55] Aggarwal BB Sethi G Baladandayuthapani V Krishnan S Shishodia S Targeting cell signaling pathways for drug discovery: an old lock needs a new key J Cell Biochem 2007 102 580 592 10.1002/jcb.21500 17668425

